# Host and viral RNA-binding proteins involved in membrane targeting, replication and intercellular movement of plant RNA virus genomes

**DOI:** 10.3389/fpls.2014.00321

**Published:** 2014-07-07

**Authors:** Kiwamu Hyodo, Masanori Kaido, Tetsuro Okuno

**Affiliations:** Laboratory of Plant Pathology, Graduate School of Agriculture, Kyoto University, KyotoJapan

**Keywords:** RNA-binding protein, RNA virus, cellular membrane, cell-to-cell movement, RNA replication

## Abstract

Many plant viruses have positive-strand RNA [(+)RNA] as their genome. Therefore, it is not surprising that RNA-binding proteins (RBPs) play important roles during (+)RNA virus infection in host plants. Increasing evidence demonstrates that viral and host RBPs play critical roles in multiple steps of the viral life cycle, including translation and replication of viral genomic RNAs, and their intra- and intercellular movement. Although studies focusing on the RNA-binding activities of viral and host proteins, and their associations with membrane targeting, and intercellular movement of viral genomes have been limited to a few viruses, these studies have provided important insights into the molecular mechanisms underlying the replication and movement of viral genomic RNAs. In this review, we briefly overview the currently defined roles of viral and host RBPs whose RNA-binding activity have been confirmed experimentally in association with their membrane targeting, and intercellular movement of plant RNA virus genomes.

## INTRODUCTION

Positive-strand RNA [(+)RNA] plant viruses are the most abundant in plant viruses, and cause diverse diseases in host plants. Because of the RNA nature of their genomes, it is not surprising that RNA-binding proteins (RBPs) of viral and host origin affect multiple steps of virus infection. It is known that viral RNA (vRNA) has critical non-template functions in addition to the essential function as a replication template (Pathak et al., 2011). After entry into the host cell, the genomic RNA of the (+)RNA virus serves as mRNA in the production of the replication proteins. The viral replication proteins recognize the viral genomic RNAs rapidly and specifically from within a pool of abundant cellular RNAs (e.g., rRNA, tRNA, and mRNA) before vRNAs are degraded by antiviral mechanisms. Plant (+)RNA viruses use organelle membranes, such as those of endoplasmic reticulum (ER), peroxisome, chloroloplast, mitochondrion, and tonoplast, by remodeling their structures for vRNA replication (Miller and Krijnse-Locker, 2008; Laliberté and Sanfaçon, 2010; Nagy and Pogany, 2011; Hyodo and Okuno, 2014). Thus, one of the critical steps in early replication is the selective recruitment of the (+)RNA template to the membrane and establishment of the viral replication complexes (VRCs).

Following the translation and replication processes, (+)RNA plant viruses spread their infection into neighboring uninfected cells through plasmodesmata (PD) by using their encoded movement protein (MP). Some plant viruses move through PD in the form of virus particles and others are thought to move in ribonucleoprotein (RNP) complexes that contain MP (Scholthof, 2005). MPs from a wide range of genera of plant viruses have been reported to bind nucleic acids in a sequence-non-specific manner (Waigmann et al., 2004; Lucas, 2006). The interaction between MPs and viral genomic RNAs is required for efficient virus cell-to-cell movement.

In this review, we overview the currently defined roles of viral- and host-derived RBPs in virus infection by focusing on the proteins whose RNA-binding activity has been confirmed experimentally in association with their membrane targeting, and intercellular movement of plant RNA virus genomes. We recommend readers consult several current reviews that discuss other topics relating to the roles of host RBPs during RNA virus infection (Li and Nagy, 2011; Huh and Paek, 2013).

## VIRAL RBPs INVOLVED IN MEMBRANE TARGETING OF VIRAL GENOMIC RNAs AND THEIR REPLICATION

*Red clover necrotic mosaic virus* (RCNMV) has a bipartite genome, RNA1 and RNA2 (Okuno and Hiruki, 2013). An interesting feature of RCNMV is that RNA1 and RNA2 use different mechanisms for template selection during replication. RNA1 encodes an auxiliary replication protein p27 and the RNA-dependent RNA polymerase (RdRp) p88^pol^. p27 and p88^pol^ localize to the ER membrane, where vRNA replication occurs (Turner et al., 2004; Kusumanegara et al., 2012; Hyodo et al., 2013). The membrane association of p27 is mediated by a stretch of 20 amino acids located in its N-terminal region that forms an amphipathic α-helix (Kusumanegara et al., 2012). p27 interacts directly with many partners such as p27 itself, p88^pol^, viral genomic RNAs, and host heat shock proteins, Hsp70 and Hsp90, and ADP ribosylation factor 1, and has multiple functions during virus infection (Mine et al., 2010, 2012; Iwakawa et al., 2011; Hyodo et al., 2013). RNA2 encodes no replication proteins, and its replication depends entirely on p27 and p88^pol^ supplied by RNA1. To recruit the replication proteins, RNA2 has a Y-shaped RNA element (YRE) in its 3′-untranslated region (UTR; An et al., 2010). An *in vitro* RNA-aptamer (Strepto-Tag) affinity and immunoprecipitation assay showed that the YRE is necessary and sufficient to interact with p27 and with the 480 kDa replication complex (Iwakawa et al., 2011). The RNA-binding activity of p27 is required for recruiting RNA2 to the membrane (Hyodo et al., 2011).

**FIGURE 1:**
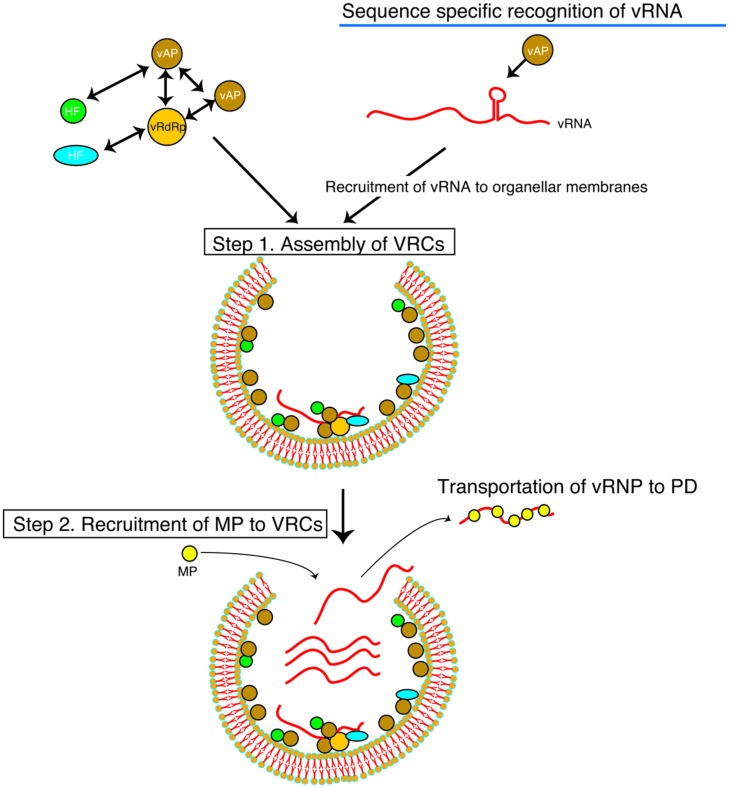
**Schematic representation of the multiplication steps of a (+)RNA virus.** Viral genomic RNA serves as the template for translation of the replicase proteins (vRdRp, viral RNA dependent RNA polymerase; vAP, auxiliary replicase protein). vAP recognizes vRNA in a sequence-specific manner and recruits to a selected membrane. vAP and vRdRp proteins interact with each other to form viral replication complexes (VRCs) with host proteins (HF) and vRNA (Step 1). Viral movement protein (MP) that is translated near the VRCs binds to vRNA. In the case of RCNMV, the MP expressed ectopically is recruited to the VRCs (Step 2; Kaido et al., 2009). MP binds to vRNA in or near the VRCs in a sequence non-specific manner and is transported to plasmodesmata (PD).

p27–p27/p88^pol^ interactions are not required for specific RNA-binding, suggesting that a monomeric form of p27 can recognize the YRE as the hallmark of RCNMV RNA2 and avoid the recruitment of unrelated RNAs from the pool of heterogeneous RNAs (**Figure 1**, Step 1). Interestingly, the p27–YRE interaction is modulated by Hsp90 (Mine et al., 2012). The p27–YRE interaction is disrupted by pharmacological inhibition of Hsp90, which also compromises the interaction between p27 and Hsp90, suggesting that Hsp90 modulates the conformation of p27 to make it suitable for recognizing YRE. This is consistent with the observations that purified recombinant p27 expressed in *Escherichia coli* has no affinity for the YRE *in vitro*, whereas p27 directly and specifically recognizes this RNA element in plant-derived cell-free extracts (Hyodo et al., 2011; Iwakawa et al., 2011; Mine et al., 2012).

RNA1 does not have YRE-like structures and is not recognized by the replication proteins supplied *in trans* (Okamoto et al., 2008; Iwakawa et al., 2011). However, both p27 and p88^pol^ interact with their ongoing translation templates *in cis* (Iwakawa et al., 2011). The RNA-binding activity of p27 is also important for the replication of RNA1 (Hyodo et al., 2011).

Replication of *Tomato bushy stunt virus* (TBSV) and related *Cucumber necrosis virus* (CNV) takes place at the peroxisomal membrane (McCartney et al., 2005; Panavas et al., 2005). TBSV and CNV infections cause the formation of peroxisomal multivesicular bodies, which may be derived from ER membranes (Barajas et al., 2009; Rochon et al., 2014). Auxiliary replication protein p33 of TBSV that has multiple functions during infection (Nagy et al., 2012) localizes to peroxisomes and plays a role in recruiting the vRNA to the peroxisome membrane. p33 has both non-specific and specific RNA-binding activities (Rajendran and Nagy, 2003; Pogany et al., 2005; Stork et al., 2011). In the specific binding mode, p33 binds to an internal replication element (IRE) located within the p92^pol^ RdRp coding region of the viral genome (Pogany et al., 2005). The absence of the p33–p33/p92 interaction domain in p33 prevents specific RNA binding but allows non-specific RNA binding, suggesting that a multimeric (possibly dimeric) form of this protein is involved in the specific recognition of the viral replication template. The C-C mismatch within a conserved RNA helix of IRE is likely to be the hallmark of the TBSV replication template because mutations that disrupt the C-C mismatch completely abolish p33 binding to the IRE and p33-mediated recruitment to replication sites (Monkewich et al., 2005; Pogany et al., 2005; Pathak et al., 2012).

RNA replication of *Brome mosaic virus* (BMV) takes place in ER membrane-associated invaginations or spherules induced by replication protein 1a (Schwartz et al., 2002). In these sites, vRNAs are strongly protected from nucleases and presumably other antiviral factors (Schwartz et al., 2002). Although direct binding between 1a and vRNAs has not been demonstrated, 1a recruits template RNAs to the ER (Chen and Ahlquist, 2000; Chen et al., 2001; Schwartz et al., 2002). The intergenic region of RNA3 contains a box B motif that is conserved with the TΨC loop of tRNAs. The same box B motif is found in the 5′-UTRs of RNAs 1 and 2. These box B motifs and flanking sequences are likely to be required for the recruitment of the BMV RNAs into the VRCs, because mutations in box B motifs disrupt 1a-mediated *in vivo* stabilization of RNA2 and RNA3 (Janda and Ahlquist, 1998; Sullivan and Ahlquist, 1999; Chen et al., 2001).

A recent publication by Kawamura-Nagaya et al. (2014) showed that a 126-kDa replication protein of *Tobacco mosaic virus* (TMV) binds the 5′-UTR of the genomic RNA cotranslationally, and that this binding plays an important role in regulation of vRNA translation and replication.

## HOST RBPs INVOLVED IN SUBCELLULAR LOCALIZATION OF VIRAL AND SUBVIRAL RNAs AND THEIR REPLICATION

Eukaryotic translation elongation factor 1A (eEF1A) is an abundant cellular protein that functions in protein degradation, apoptosis, nucleocytoplasmic trafficking, heat shock, and organization of the actin cytoskeleton in addition to its canonical role in delivering aminoacyl-tRNA to the elongating ribosome (Mateyak and Kinzy, 2010). Several RNA viruses have been reported to use eEF1A for RNA replication by a variety of mechanisms (Li et al., 2013). Studies of TBSV have provided information about the eEF1A function in viral template recruitment. A replication silencer element (RSE) located within the 3′-UTR of TBSV (+)RNA is the binding site of eEF1A (Li et al., 2009). The RSE is a *cis*-acting RNA element essential for TBSV replicase complex assembly and (–)RNA synthesis (Pogany et al., 2003; Panaviene et al., 2005). Pharmacological inhibitors of eEF1A, such as didemnin B (DB) and gamendazole (GM), block eEF1A–vRNA interaction, inhibit the membrane association of the TBSV-derived RNA replicon, and repress vRNA synthesis *in vitro* (Li et al., 2010). These findings suggest that eEF1A stimulates the recruitment of the (+)RNA template to the site of replication. Consistent with this, neither DB nor GM inhibits the RNA synthesis on the pre-assembled viral replicase complex (Li et al., 2010).

*Bamboo mosaic virus* (BaMV) RNA has been detected in chloroplasts, a putative replication site of BaMV (Lin et al., 1993). Chloroplast phosphoglycerate kinase (chl-PGK) of *Nicotiana benthamiana* can bind to the 3′-UTR of BaMV (+) RNA (Lin et al., 2007). PGK is an ATP-generating enzyme that acts in the glycolytic, gluconeogenesis, and photosynthetic pathways. chl-PGK is a nuclear-encoded protein that possesses an N-terminal targeting signal, which mediates posttranslational protein transport into the chloroplast (Jarvis and López-Juez, 2013). The role of chl-PGK during BaMV infection was reported recently (Cheng et al., 2013). In protoplasts derived from chl-PGK-silenced *N. benthamiana*, the accumulation of BaMV coat protein (CP) is inhibited, suggesting that PGK is required for the replication of BaMV. Interestingly, overexpression of cytosolic- or nuclear-localized PGK mutants has detrimental effects on BaMV infection. This is consistent with the author’s prediction that these PGK mutants mislead BaMV RNA away from chloroplasts to the cytoplasm or the nucleus that are unsuitable sites for viral replication. BaMV seems to use the trafficking system of chl-PGK to target vRNA to the chloroplasts for replication. In support of this, artificial chloroplast-targeting EF1a, which binds to BaMV (+)RNA, can partially complement BaMV accumulation in chl-PGK-knockdown *N. benthamiana* (Lin et al., 2007; Cheng et al., 2013). BaMV RNA localization is detected throughout the chloroplasts, suggesting that BaMV does not simply associate with the chloroplast outer membrane but that it enters the stroma (Lin et al., 1993; Cheng et al., 2013). It will be interesting to investigate the possible involvement of the chloroplast translocation machinery in BaMV localization.

A recent publication by Chaturvedi et al. (2014) showed that a tomato bromodomain-containing host protein (BRP1, also known as VIRP1) binds a satellite RNA of *Cucumber mosaic virus* (CMV), and that BRP1 plays an important role in the nuclear importation and multiplication of the satellite RNA. BRP1 also binds *Potato spindle tuber viroid* RNA both *in vitro* and *in vivo*, and is involved in the nuclear transportation and multiplication of the viroid (Martínez de Alba et al., 2003; Kalantidis et al., 2007). Further studies on host RBPs are awaited to elucidate the replication and movement mechanism of these subviral pathogens.

## VIRAL RBPs INVOLVED IN THE INTERCELLULAR MOVEMENT OF vRNAs

Plant RNA viruses encode MPs that play a central role in the transport of viral genomes into neighboring uninfected cells. CPs also play ancillary roles in the virus cell-to-cell movement of some viruses (see reviews by Waigmann et al., 2004; Lucas, 2006; Harries and Nelson, 2008; Harries et al., 2010; Peña and Heinlein, 2012). Viruses that do not require virion formation for cell-to-cell movement are thought to form viral RNPs (vRNP) that contain viral genomic RNA and MP for the transport to and through PD. TMV MP, the first MP shown to bind nucleic acids, can bind both single-stranded RNA and DNA in a sequence-non-specific and cooperative manner, but cannot bind double-stranded DNA (Citovsky et al., 1990, 1992). After the first report of TMV MP binding to nucleic acids, the binding of other MPs to nucleic acids has been reported for 26 other viruses, including (+)RNA viruses, (-) RNA viruses, and DNA viruses (reviewed by Waigmann et al., 2004). In the past decade, at least nine MPs have been added to the list of nucleic acid-binding MPs: *Cowpea mosaic virus* (CPMV; Carvalho et al., 2004); *Prunus necrotic ringspot virus* (PNRSV; Herranz and Pallás, 2004); *Rice yellow stunt virus* (Huang et al., 2005); *Apple chlorotic leaf spot virus* (Isogai and Yoshikawa, 2005); *Rice dwarf virus* (Ji et al., 2011); *Parietaria mottle virus* (Martínez et al., 2014); *Pelargonium flower break virus* (Martínez-Turiño and Hernández, 2011); *Melon necrotic spot virus* (Navarro et al., 2006); *Rice stripe virus* (Xiong et al., 2008). Most MPs can bind single-stranded nucleic acids, and only three viral MPs (one geminivirus and two hordeiviruses) have been shown to bind double-stranded nucleic acids (Bleykasten et al., 1996; Donald et al., 1997; Rojas et al., 1998).

One significant function of RNA-binding by MP is to protect vRNA from the degradation by RNAse. For example, the binding of CMV MP to vRNA is rather weak and is RNAse-sensitive (Li and Palukaitis, 1996). CMV uses its CP to strengthen the weak binding to form RNAse-resistant vRNP complexes used in cell-to-cell movement (Nagano et al., 2001; Andreev et al., 2004; Kim et al., 2004). Triple gene block protein 1 (TGBp1) of *Poa semilatent virus* (PSLV) forms a high concentration salt-resistant vRNP (Kalinina et al., 2001), which may confer the ability for cell-to-cell and systemic movement in a CP-independent manner. The importance of the RNA-binding ability of MPs in viral cell-to-cell movement was confirmed by mutational analysis of the RNA-binding domains of RCNMV and PNRSV MPs (Giesman-Cookmeyer and Lommel, 1993; Herranz et al., 2005). The RNA-binding domains of PSLV MP (TGBp1) are unnecessary for cell-to-cell movement but are required for the systemic movement of the virus (Kalinina et al., 2001). Interestingly, the salt-resistant tight binding of *Turnip crinkle virus* (TCV) MP is a restricting factor for systemic infection by the virus (Wobbe et al., 1998). TCV strain B has a greater ability to spread in *Arabidopsis thaliana* ecotype Di-17 compared with TCV strain M. The two MPs differ only by one amino acid, and the binding to RNA by the MP of strain M has a greater tolerance to high salt concentration compared with that by the MP of strain B. It is possible that improper dissociation of the MP of strain M from vRNA can block the viral proliferation steps in newly infected cells.

It is interesting that CPMV MP can bind to the single-stranded nucleic acids despite the fact that CPMV moves through PD in the form of virions (van Lent et al., 1990; Wellink et al., 1993). The formation of a MP-vRNA complex of CPMV may be required for systemic infection, or CPMV MP may have distinct roles other than the movement of virions through PD.

All MPs that bind to nucleic acids reported so far do not exhibit sequence specificity. This raises the question of how MPs find vRNAs in infected cells. It is noteworthy that MPs of several RNA viruses, including TMV (Heinlein et al., 1998; Asurmendi et al., 2004; Kawakami et al., 2004), *Potato virus X* (PVX; Bamunusinghe et al., 2009; Tilsner et al., 2012, 2013), BMV (Dohi et al., 2001), and RCNMV (Kaido et al., 2009), colocalize with their VRCs. This may enable MPs to attach to viral genomes in the vicinity (**Figure 1**, Step 2). RCNMV MP is recruited to the VRC through the interaction with a host protein contained in the VRC (Kaido et al., 2009, 2011; Kaido and Okuno, unpublished results).

The only reported example of RNA *cis*-element that is required for virus cell-to-cell movement is the 5′-terminal stem–loop structure (SL1) of PVX genomic RNA. The green fluorescent protein gene containing the 107 nt terminal sequence of the PVX 5′-UTR was transported effectively to neighbor cells by cobombarded PVX (Lough et al., 2006). Immunoelectron microscopy and atomic-force microscopy revealed that the 5′-proximal region of PVX RNA is recognized by its CP, and that PVX MP (TGBp1) interacts with the CP (Karpova et al., 2006; Zayakina et al., 2008). Because PVX does not require virion formation for its cell-to-cell movement (Lough et al., 2000), PVX seems to move between cells through the CP-mediated TGBp1–vRNA complex.

## HOST RBPs INVOLVED IN THE INTERCELLULAR MOVEMENT OF vRNAs

In addition to MPs, plant viruses are thought to use host proteins for the cell-to-cell and systemic movement of viral genomes. To date, more than 40 cellular proteins have been reported to interact with MPs and some of these have been identified as a host factor involved in a variety of processes related to virus movement (Harries and Ding, 2011; Niehl and Heinlein, 2011). However, the RNA-binding activity of these host proteins remains to be defined.

Using two-dimensional electrophoresis, northwestern blot analysis, and mass spectrometry, 24 proteins of *N. benthamiana* were identified as being able to interact with the PVX 5′-terminal SL1 sequence (Cho et al., 2012b). Of these, two RBPs have been reported to be involved in the movement of a plant virus. NbDnaJ protein was shown by an electro-mobility shift assay to interact specifically with the minus-strand SL1 *in vitro*. NbDnaJ also interacts with PVX CP *in planta*, and negatively affects the systemic movement of the virus (Cho et al., 2012c). NbMPB2Cb, another SL1-interacting protein, interacts with PVX CP, TGBp1, and TGBp2 *in planta*, and negatively regulates the systemic movement of PVX (Cho et al., 2012a). NbMPB2Cb changed its subcellular localization from microtubules to ER in association with PVX infection. NbMPB2Cb may disturb the formation of TGBp2-induced ER-derived vesicles and this may negatively affect the functions of the vesicles by RNA–protein or protein–protein interactions.

## PERSPECTIVES

Viral and host RBPs play diverse critical roles during infection. Some RBPs recruit vRNAs from the cytosol to the membranous replication sites, and others pick up and deliver RNAs to neighboring cells. These RBPs may require partners to accomplish their roles, and these partners may differ, depending on the infection stage; translation, replication, intra-, and inter-cellular movement. Viral replication proteins, such as RCNMV p27 and TBSV p33, are multifunctional proteins that interact with viral and host proteins in addition to vRNAs and play central roles in virus infection. It is also noteworthy that, during infection by (+)RNA viruses, translation, replication, and intra- and intercellular movements are often closely linked (Tilsner and Oparka, 2012). To investigate the regulatory mechanisms underlying these processes, we need to identify more RBPs and their partners. The recently developed interactome approach comprising *in vivo* UV cross-linking, capture of polyadenylated RNA on oligo(dT)-coated beads, and release of bound proteins by nuclease digestion has successfully identified a number of RBPs that bind specifically to cellular mRNAs (Baltz et al., 2012; Castello et al., 2012; Mitchell et al., 2013). Application of this approach combined with the RNA aptamer pulldown assay will be useful for identifying candidate RBPs involved in membrane targeting, intracellular trafficking and intercellular movement of plant RNA virus genomes.

## Conflict of Interest Statement

The authors declare that the research was conducted in the absence of any commercial or financial relationships that could be construed as a potential conflict of interest.
